# Evidence that involucrin, a marker for differentiation, is oxygen regulated in human squamous cell carcinomas

**DOI:** 10.1038/sj.bjc.6601585

**Published:** 2004-02-03

**Authors:** S-C Chou, Y Azuma, M A Varia, J A Raleigh

**Affiliations:** 1 Department of Radiation Oncology, UNC School of Medicine, CB 7512, Chapel Hill, NC 27599, USA

**Keywords:** hypoxia, differentiation, pimonidazole, involucrin, oxygen regulation

## Abstract

The majority of hypoxic cells in squamous cell carcinomas of the head and neck and cervix express involucrin, a molecular marker for differentiation. This raises the question of whether involucrin is an oxygen-regulated protein and, if so, whether it could serve as an endogenous marker for tumour hypoxia. Consistent with oxygen regulation, involucrin protein was found to increase with increasing hypoxia in confluent cultures of moderately differentiated human SCC9 cells. Cells harvested at the point of confluence and exposed to graded concentrations of oxygen revealed a *K*_m_ of approximately 15 mmHg for involucrin induction. This is similar to *K*_m_s for HIF-1*α*, CAIX and VEGF. Involucrin induction showed a steep dependence on *p*O_2_ with a transition from minimum to maximum expression occurring over less than an order of magnitude change in *p*O_2_. In contrast to SCC9 cells, involucrin was not induced by hypoxia in poorly differentiated SCC4 cells. It is concluded that involucrin is an oxygen-regulated protein, but that differentiation modulates its transcription status with respect to hypoxia induction.

Hypoxia is associated with poor prognosis in squamous cell carcinomas affecting both local control and distant spread (Hockel *et al*., 1996a, 1996b, 1999; Nordsmark *et al*, 1996; Fyles *et al*, 2002; Kaanders *et al*, 2002). Local control is believed to depend on local radiation response while distant spread is thought to depend, at least in part, on the induction of oxygen-regulated proteins. In order to test this, pimonidazole, an extrinsic marker for tissue hypoxia (Arteel *et al*, 1995; Kennedy *et al*, 1997; Varia *et al*, 1998; Raleigh *et al*, 1999), with prognostic value (Kaanders *et al*, 2002) was used to examine whether ORPs such as VEGF (Raleigh *et al*, 1998a), metallothionein (Raleigh *et al*, 2000), HIF-1*α* (Janssen *et al*, 2002), Glut-1 (Airley *et al*, 2003) and CAIX (Olive *et al*, 2001) were, in fact, associated with cellular hypoxia in human tumours. Unexpectedly, VEGF and metallothionein (MT) were not expressed in the majority of hypoxic cells in squamous cell carcinomas (Raleigh *et al*, 1998a, 2000) even though these ORPs were induced by hypoxia in experimental systems (Shweiki *et al*, 1992; Raleigh *et al*, 1998b; Murphy *et al*, 1999).

A possible explanation for this apparent anomaly was found in reports that VEGF and MT are expressed in oxygenated basal lamina of normal stratified epithelia and not in more differentiated, suprabasal layers farthest from the blood vessels (Quaife *et al*, 1994; Sundelin *et al*, 1997; Viac *et al*, 1997). This led to the conclusion that VEGF and MT are downregulated by differentiation in stratified epithelia (Quaife *et al*, 1994; Viac *et al*, 1997). In analogy with their untransformed counterpart, squamous cell carcinomas often express markers for terminal differentiation in the centre of tumour nests farthest from blood vessels (Roland *et al*, 1996). Pimonidazole binding was known to occur in these regions (Kennedy *et al*, 1997), and subsequent studies demonstrated that the majority of the hypoxic cells express involucrin, a molecular marker for epithelial cell differentiation (Raleigh *et al*, 2000). It was concluded, therefore, that the lack of VEGF and MT expression in hypoxic cells was due to downregulation by differentiation. At the same time, it appeared that involucrin might be an oxygen-regulated protein.

Involucrin is a 96 kDa cell envelope protein that appears in free form in the early stages of keratinocyte terminal differentiation. During the late stages of differentiation, involucrin is crosslinked with proteins and lipids to form cornified cell envelopes in the uppermost cells of stratified epithelia (Eckert and Welter, 1996). Five AP-1 consensus sites exist in the promoter region of the involucrin gene with two sites accounting for 80% of promoter activity (Crish *et al*, 2002). One site is proximal while the other is distal to the transcription start site. The proximal site is regulated via a mitogen-activated protein kinase pathway that includes PKC, Ras, MEKK1, MEK3 and p38/RK (Efimova *et al*, 1998). Gel supershift analyses show that *jun*B, *jun*D and *Fra-*1 are the major AP-1 transcription factors regulating involucrin expression (Eckert and Welter, 1996). However, cotransfection of involucrin promoter constructs with c-*jun* and c-*fos* can increase involucrin promoter activity, indicating that c-Jun also stimulates involucrin transcription (Takahashi and Iizuka, 1993; Efimova *et al*, 1998). Although involucrin had not been identified previously as an ORP, c-Jun/AP-1 is known to be responsive to hypoxia in squamous cell carcinoma cells (Bandyopadhyay *et al*, 1995; Laderoute *et al*, 2002) and it was conceivable that involucrin expression was oxygen regulated.

The present investigation examines whether involucrin is oxygen regulated in an *in vitro* model comprising moderately differentiated SCC9 and poorly differentiated SCC4 squamous cell carcinoma cells (Rheinwald and Beckett, 1980, 1981). The model was of interest because Rice *et al* (1988) had shown that involucrin increases spontaneously in postconfluent cultures of SCC9 cells. Hypoxia is generated in unstirred, high-density cell cultures (Boag, 1969; Whillans and Rauth, 1980; Jones, 1985; Kaluz *et al*, 2002) and, although other explanations are possible, it seemed that involucrin might be induced by hypoxia in the SCC9 cultures.

Investigations of potentially useful endogenous markers of hypoxia such as CAIX and Glut-1 have shown that immunostaining for these proteins extends beyond the edges of pimonidazole binding (Olive *et al*, 2001; Kaanders *et al*, 2002; Airley *et al*, 2003). Many oxygen-regulated processes are half maximally induced at oxygen partial pressures (*K*_m_=6–20 mmHg) (Leith and Michelson, 1995; Jiang *et al*, 1996; Chiarotto and Hill, 1999; Wykoff *et al*, 2000) that would strongly inhibit the binding of nitroimidazole hypoxia markers such as pimonidazole, EF5 and misonidazole (*K*_m_=0.8–2.0 mmHg) (Franko *et al*, 1987; Arteel *et al*, 1995; Gross *et al*, 1995; Koch *et al*, 1995). This could account for the immunostaining patterns for Glut-1 and CAIX. If involucrin were also induced in the range of 6–20 mmHg, it might be expressed in tumour microregions that did not bind detectable levels of pimonidazole. In order to explore this possibility, the *K*_m_ for involucrin expression has been measured in suspension cultures of SCC9 cells.

In poorly differentiated squamous cell carcinomas, involucrin immunostaining is generally weak even in tumour regions that avidly bind pimonidazole (Azuma *et al*, 2003). This would appear to be inconsistent with oxygen regulation. However, the transcription status of oxygen-regulated genes can be coregulated by differentiation (Webster *et al*, 1990; Claffey *et al*, 1992; Quaife *et al*, 1994; Levy and Kelly, 1997; Viac *et al*, 1997) and the effect of differentiation on involucrin expression was therefore examined by comparing its expression in moderately differentiated SCC9 and poorly differentiated SCC4 cells exposed to acute and chronic hypoxia.

## MATERIALS AND METHODS

### Chemicals

The hypoxia marker, pimonidazole hydrochloride (Hypoxyprobe™-1; Chemicon International Inc., Temecula, CA, USA), was used as previously described (Arteel *et al*, 1995; Kennedy *et al*, 1997; Varia *et al*, 1998; Raleigh *et al*, 1999, 2000). 4-Nitrophenyl phosphate (alkaline phosphatase substrate), phosphate-buffered saline (PBS) pellets, foetal bovine serum and hydrocortisone (cat # H-0396) were obtained from Sigma (St Louis, MO, USA). Liquid 3,3′-diaminobenzidine (DAB) peroxidase substrate was obtained from DAKO Corp (Carpinteria, CA, USA). Aqueous 2% formalin was obtained from Polysciences, Inc. (Warrington, PA, USA). Enzyme-grade polyoxyethylene ether (Brij 35), polyoxyethylenesorbitan monolaurate (Tween 20), tris(hydroxymethyl)aminomethane (Tris), Biomeda Crystal/Mount, ProbeOn Plus glass slides and miscellaneous reagent-grade chemicals were obtained from Fisher Scientific Company (Norcross, GA, USA). Aqua Haematoxylin was obtained from Innovex Biosciences (Richmond, CA, USA). Gas tanks containing certified quantities of oxygen and 5% CO_2_ balanced with nitrogen were purchased from National Welders Supply Company, Inc. (Raleigh, NC, USA).

### Immunological reagents

Supernatant from hybridoma clone 4.3.11.3 containing antipimonidazole IgG_1_ monoclonal antibody at a concentration of 70 *μ*g ml^−1^ (Chemicon International Inc., Temecula, CA, USA) was used for the immunohistochemical detection of protein adducts of reductively activated pimonidazole as described previously (Arteel *et al*, 1995; Kennedy *et al*, 1997; Varia *et al*, 1998). Diluted aliquots of rabbit polyclonal antipimonidazole antisera were used for the enzyme-linked immunosorbent assay (ELISA) of pimonidazole binding to cell lysates (Arteel *et al*, 1995). A biotin-conjugated F(ab′)_2_ fragment of a rabbit anti-mouse IgG was obtained from Accurate Chemical Scientific Corp. (Westbury, NY, USA) and used as the secondary reagent for the immunohistochemical detection of pimonidazole binding. Protein blocker and peroxidase-conjugated streptavidin were obtained from DAKO Corp. An IgG_1_ mouse anti-human involucrin antibody clone SY5 used for the immunohistochemical detection of involucrin was obtained from Sigma. An ELISA kit containing rabbit anti-human involucrin antisera and affinity-purified goat anti-rabbit IgG conjugated to alkaline phosphatase used to detect involucrin in cell lysates were obtained from Biomedical Technologies Inc. (Stoughton, MA, USA).

### Confluent cell culture

SCC9 and SCC4 cell lines derived from a squamous cell carcinoma of the human tongue (American Type Culture Collection, Rockville, MD, USA) were grown in Dulbecco's modified eagle medium/F12 containing 1.0 mM.calcium ion concentration and supplemented with 10% foetal bovine serum, 0.4 *μ*g ml^−1^ of hydrocortisone and 14 mM of sodium bicarbonate. Cells were seeded at a density of 3 × 10^5^ cells in 100-mm diameter culture dishes. Every 3 days, the culture medium was exchanged with fresh medium containing 100 *μ*M pimonidazole hydrochloride as hypoxia marker. Cell samples were harvested at 1, 4, 6, 9 and 12 days after confluence. Harvested cells were washed three times with cold PBS and cell densities were measured by cytometry. Cells were lysed in cold buffer containing 0.2 mM EDTA, 10 mM Tris, 0.5% Triton X-100 and 200 *μ*l/10^6^ cells of proteinase inhibitors (1.0 *μ*g ml^−1^ of Leupeptin, 1.0 *μ*g ml^−1^ of pepstatin and 1 mM phenylmethylsulphonyl fluoride) (Gaido and Maness, 1994). Cell lysates were stored at –80°C until they were analysed by ELISA for pimonidazole adducts and involucrin.

### Immunostaining confluent cultures for involucrin and pimonidazole adducts

SCC9 cells were added to a six-well tissue culture plate at a density of 10^5^ cells per well. Each well contained four 22 mm square cover glass slides to which the cells attached. At 2 days prior to confluence, at confluence and 5 days after confluence, cells on the glass slides were fixed with 2% of formaldehyde in PBS for 20 min. The slides were washed and permeabilised with 0.02% of saponin in PBS containing 5% of serum-free protein block for 30 min. Fixed cells were incubated with antipimonidazole IgG_1_ monoclonal antibody 4.3.11.3 (1 : 50) and anti-human involucrin monoclonal antibody (1 : 100) for 1 h. The cells were then incubated with biotin-conjugated rabbit anti-mouse F(ab′)2 IgG antibody (1 : 500) for 30 min . The cells were incubated with streptavidin-conjugated peroxidase for 20 min and colour developed by incubation with DAB for 10 min. The cells were counterstained with haematoxylin at room temperature for 25 s and washed. The cover slides were placed on a microscope slide, with the cells facing the surface of the microscope slide and mounted with CrystalMount.

### Exposure of cells to hypoxia in suspension culture

When SCC9 and SCC4 cells reached confluence they were trypsinised and collected. Aliquots of 5 × 10^6^ cells in 25 ml of culture medium containing 100 *μ*M pimonidazole hydrochloride and 1.0 mM calcium ion were added to 250 ml glass vessels fitted with PTFE inlet and outlet stopcocks and a small diameter injection port (cat.# 7401–50; Ace Glass, Inc., Vineland, NJ, USA). In order to minimise cell adhesion, the vessels were silanised by treatment with Sigmacote (Sigma, St Louis, MO, USA) followed by extensive washing with distilled water. Cells were kept in suspension by attaching the gassing vessel to the deck of an orbital shaker (Model SS110504; Integrated Separation Systems, Natick, MA, USA) in a warm room maintained at 37°C. The system was flushed for 20 min in order to remove oxygen dissolved in nonglass components of the system. These included two PTFE stopcocks, a small red rubber septum port, PTFE unions connecting gas wash bottles to nylon transmission tubing (12723 Universal Connector; Ace Glass, Inc.), short lengths of flexible tygon tubing that connected reciprocating glass tubes to stiff, low-permeability 3/16 inch internal diameter nylon transmission tubing (A-06489-06; Cole-Palmer Instrument Co., Vernon Hills, IL, USA) and 150 ml of distilled water in a gas wash bottle used to humidify the gas stream. Following flushing, the system was subjected to 12 rounds of partial vacuum followed by pressurisation with gas phases containing 10, 100, 500, 5000, 10 000, 20 000 or 25 000 ppm oxygen and 5% CO_2_ balanced with nitrogen. The gas exchanges – which were carried out over a period of 5 min – facilitated the rapid equilibration of molecular oxygen in gas and aqueous phases. Once equilibrated, cells were incubated with shaking under a continuous flow of gas. Previous studies showed that cell viability is not affected by this procedure (Arteel *et al*, 1995). Teflon and nylon have low oxygen permeability (see Cole-Palmer Instrument Company 2003/04 catalogue, p.1910) and once flushed were not expected to be a source of oxygen contamination. Rubber and tygon are more permeable, but oxygen contamination from rubber septa and short lengths of tygon tubing was also considered to be insignificant in a system equilibrated and then continuously flushed with a flow of gas. Whillans and Rauth (1980) have shown that continuous flushing following equilibration is adequate to control pO_2_ down to at least 0.01% even when relatively long sections of tygon tubing are used.

The *K*_m_ experiment was repeated twice and the data points averaged for both pimonidazole binding and involucrin expression. Control experiments showed that the presence of pimonidazole did not affect involucrin expression. Cells were collected, washed three times with cold PBS and cell densities were measured by cytometry. Cells were lysed and stored at –80°C for subsequent ELISA analysis for involucrin and pimonidazole adducts.

### ELISA

The ELISA for pimonidazole adducts followed a previously published method for 2-nitroimidazole hypoxia markers (Raleigh *et al*, 1994; Thrall *et al*, 1994; Arteel *et al*, 1995), except that cell lysates were prepared by homogenisation without pronase K digestion. Briefly, 100 *μ*l well^−1^ of serial dilutions of cell lysates and serial dilutions of pimonidazole hydrochloride standards were incubated for 1 h at 37°C in 96-well microtitre plates containing 100 *μ*l well^−1^ of rabbit polyclonal antipimonidazole antisera diluted 6 : 10 000 in PBS-Tween (0.05% Tween 20 in PBS). The mixtures were transferred to ELISA plates coated with a Ficoll-pimonidazole conjugate as solid-phase antigen and the plates incubated for 1 h at 37°C. The plates were washed with PBS-Tween by means of an Ultrawash plate washer (Dynex Technologies Inc., Chantilly, VA, USA); 100 *μ*l well^−1^ of a 1 : 2000 goat anti-rabbit antibody conjugated with alkaline phosphatase was added, and the plates were incubated for 1 h at 37°C. The plates were washed and 100 *μ*l well^−1^ of a 1 mg ml^−1^ solution of alkaline phosphatase substrate dissolved in 10% diethanolamine pH 9.8 buffer was added. Colour development at 405 nm was followed for 5 min by means of a Molecular Devices plate reader. Kinetic data were analysed by means of Vmax DeltaSoft 3 software (Biometallics, Inc., Princeton, NJ, USA). ELISA data were corrected for the fact that pimonidazole hydrochloride, although a convenient standard, is 25 less effective as a competitive inhibitor than protein adducts of pimonidazole (Arteel *et al*, 1995). The data were normalised to cell lysate protein content as measured by the Bio-Rad Dc protein assay (Bio-Rad, Hercules, CA, USA) using bovine serum albumin as a standard.

The involucrin ELISA kit was used according to the directions provided by Biomedical Technologies Inc. In the final step, a secondary goat anti-rabbit IgG-conjugated alkaline phosphatase was used to detect the binding of the antiinvolucrin rabbit antisera to involucrin solid phase antigen. End point colour development at 405 nm associated with the hydrolysis of 4-nitrophenyl phosphate was recorded after 30 min. ELISA data for involucrin were normalised for protein content in the cell lysates using bovine serum albumin as a standard.

### Clinical samples

Contiguous tumour sections immunostained for involucrin and pimonidazole binding were available from head and neck squamous cell carcinomas from an earlier study (Raleigh *et al*, 2000). The study had received local Institutional Review Board approval for the type of experiment described here. Patients enrolled in the study had signed informed consent forms prior to their participation in the study (Raleigh *et al*, 2000). The Hypoxyprobe-1 used for the clinical studies was obtained from NPI, Incorporated (Belmont, MA, USA).

## RESULTS

### Involucrin and hypoxia in confluent cultures of SCC9 and SCC4 cells

**Figure 1:**
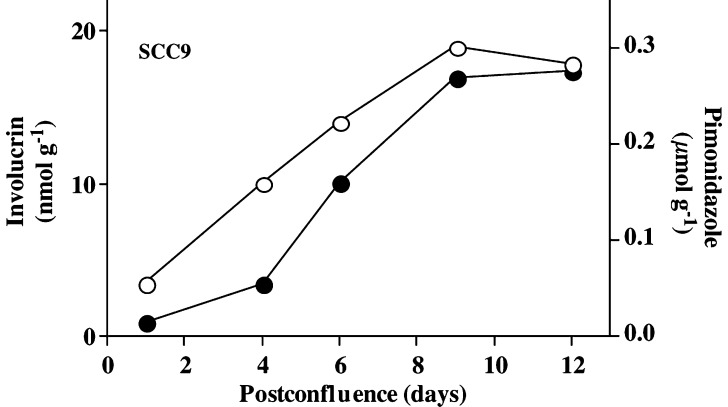
Involucrin expression (solid circles) and pimonidazole binding (open circles) in moderately differentiated SCC9 cells growing in confluent culture in the presence of 1.0 mM calcium ion. The increase in involucrin expression more or less parallels that for pimonidazole binding. The involucrin data are similar to those reported by others (Rice *et al*, 1988).

**Figure 2:**
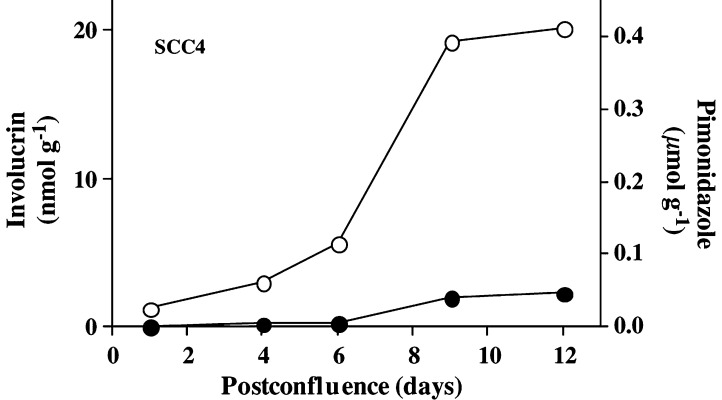
Involucrin expression (solid circles) and pimonidazole binding (open circles) in poorly differentiated SCC4 cells growing in confluent culture in the presence of 1.0 mM calcium ion concentration. Little or no involucrin is induced in the cultures, even though pimonidazole binding indicates the presence of hypoxia in the cultures.

ELISA measurements revealed a steady increase in involucrin expression in SCC9 cells beginning ca 4 days postconfluence. The increase in involucrin expression was associated with an increase in pimonidazole binding in the cultures. The maximum involucrin protein expression occurred ca 9 days postconfluence (Figure 1

). Pimonidazole binding increased in confluent cultures of SCC4 cells but, unlike SCC9 cells, a corresponding increase in involucrin did not occur (Figure 2

).

Immunostaining of postconfluent SCC9 cell cultures showed punctate patterns for both involucrin expression and pimonidazole binding. That is, the whole culture was not hypoxic but rather subsets of cells within the cultures formed pimonidazole adducts (data not shown). Owing to piling up in the cultures, it has not been possible to determine whether pimonidazole-positive cells are also involucrin positive by microscopic examination of dual stained slides (data not shown).

### *K*_m_ for involucrin expression in suspension cultures of SCC9 cells

**Figure 3:**
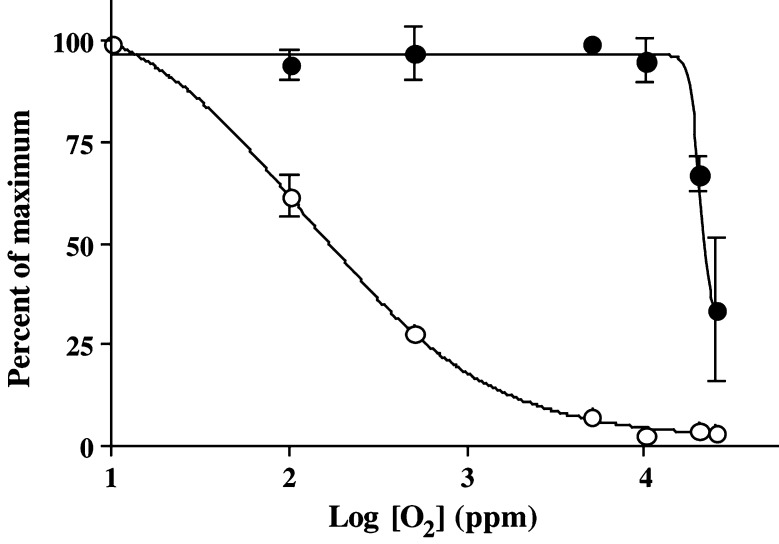
*K*_m_ curves for involucrin (solid circles) and pimonidazole binding (open circles) in SCC9 cells exposed for 2 h to different *p*O_2_ in the presence of 1.0 mM calcium ion concentration. The data represent averages of two independent experiments. Range of data for the two experiments is shown where it exceeded the dimension of the data symbol. Note the 40-fold difference in *K*_m_ between involucrin induction and pimonidazole binding and the steep *p*O_2_ dependence for involucrin induction.

Involucrin induction was half maximal at a gas phase oxygen concentration of approximately 20 000 ppm for SCC9 cells harvested at the point of confluence and exposed to graded concentrations of oxygen for 2 h (Figure 3

). A gas phase concentration of 20 000 ppm is equivalent to a partial pressure of 15 mmHg, a gas phase concentration of 2% or a dissolved oxygen concentration of 21 *μ*M at 37°C. Cells harvested 9 days postconfluence, when involucrin expression was at a maximum, showed no additional induction of involucrin during acute exposure to hypoxia in suspension culture. As was the case in confluent cultures, involucrin was not induced in SCC4 cells during hypoxic exposure in suspension culture.

The *K*_m_ for involucrin induction was ca 40 times higher than that for pimonidazole binding in SCC9 cells (Figure 3). Furthermore, the oxygen dependence for involucrin expression in SCC9 cells was much steeper than that for pimonidazole binding, increasing from minimum to maximum over less than one order of magnitude change in *p*O_2_ compared to pimonidazole binding that rose from minimum to maximum over two orders of magnitude as expected for a competition between pimonidazole and oxygen for reducing equivalents (Arteel *et al*, 1998).

### Immunostaining patterns for involucrin and hypoxia in head and neck squamous cell carcinomas

Figure 4

**Figure 4:**
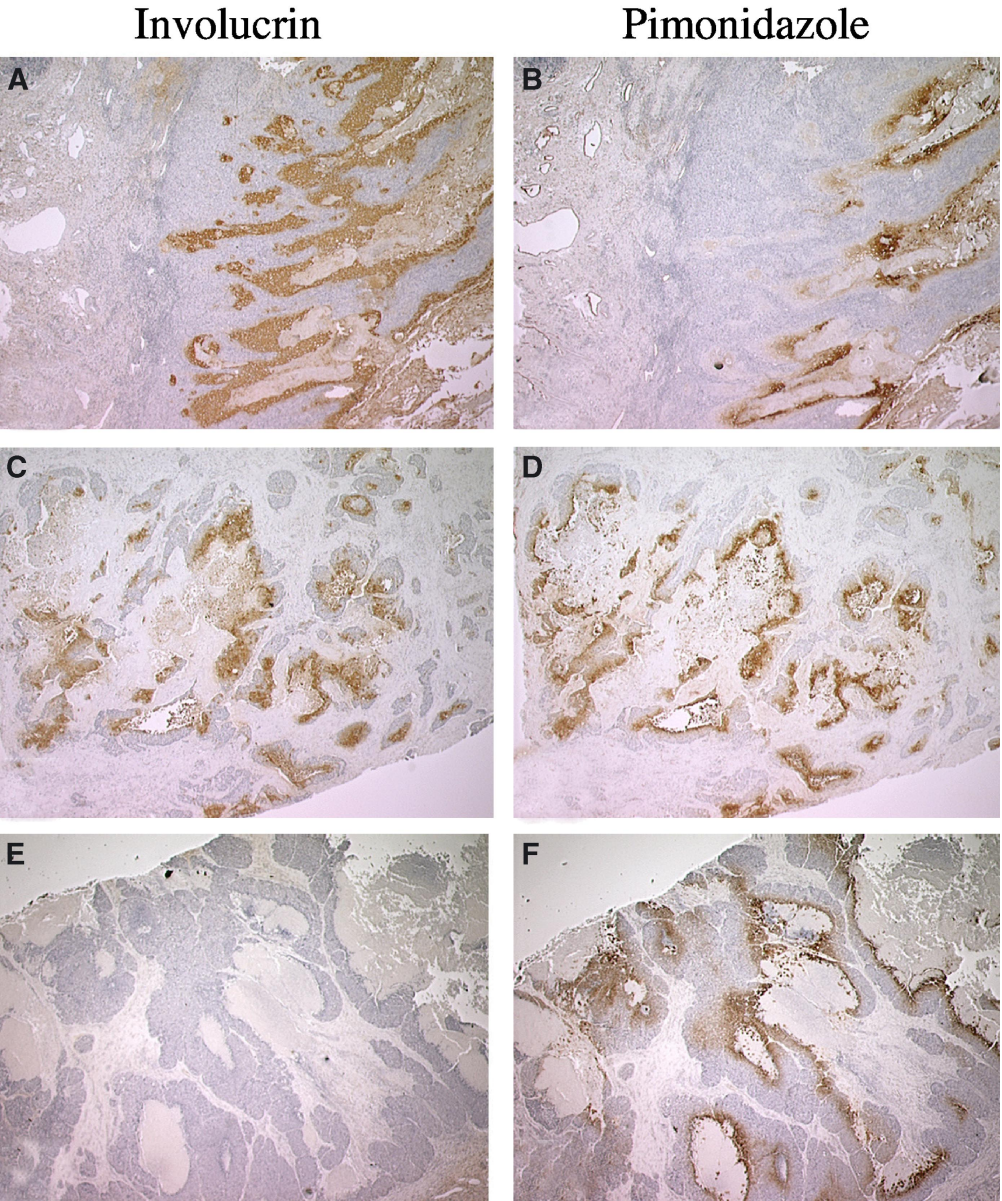
Immunostaining patterns for involucrin expression (left panels) and pimonidazole binding (right panels) in contiguous sections from squamous cell carcinomas (SCC) of the head and neck. (**A** and **B**) Immunostaining for involucrin and pimonidazole adducts, respectively, in sections from a Grade 1 floor of the mouth SCC. (**C** and **D**) Immunostaining for involucrin and pimonidazole adducts in sections from a Grade 2 larynx SCC. Immunostaining for involucrin (**A**) extends well beyond that for pimonidazole binding (**B**) and is expressed in the absence of pimonidazole binding in some microregions. (**C** and **D**) Immunostaining for involucrin and pimonidazole adducts is closely matched with the extent of involucrin immunostaining covering an area ca 1.5 that for pimonidazole. (**E** and **F**) Immunostaining for involucrin and pimonidazole adducts in sections from a Grade 3 larynx SCC. Little or no involucrin expression is observed in the presence of extensive pimonidazole binding. Original magnification: × 12.5.

shows representative examples of immunostaining for involucrin expression and pimonidazole binding in contiguous sections taken from squamous cell carcinomas of the head neck. Figure 4A and B are derived from a well-differentiated (Grade 1) squamous cell carcinoma of the floor of the mouth. In this case, immunostaining for involucrin extends well beyond the edges of immunostaining for pimonidazole adducts and, in some regions, involucrin is expressed in the absence of pimonidazole binding. Figure 4C and D are derived from a moderately differentiated (Grade 2) tumour of the larynx. In this case, the extent of immunostaining for involucrin conforms more closely to that for pimonidazole adducts, with the extent of immunostaining differing by a factor of only ca 1.5. Figure 4E and F are derived from a poorly differentiated (Grade 3) squamous cell carcinoma of the larynx. In this case, little or no involucrin is expressed even in the presence of substantial amounts of pimonidazole binding.

## DISCUSSION

Involucrin expression increases with increasing hypoxia in confluent cultures of SCC9 cells consistent with the idea that involucrin is an oxygen-regulated protein. The induction of involucrin during hypoxic exposure of SCC9 cells in suspension culture confirms that involucrin can be induced by hypoxia in moderately differentiated squamous cell carcinoma cells. Induction occurs over a period of 2 h and is, therefore, relatively rapid. In contrast to SCC9 cells, hypoxia induces little or no involucrin in poorly differentiated SCC4 cells in spite of the fact that the involucrin gene is reported to be functional with ample quantities of involucrin mRNA present in these cells (Gibson *et al*, 1996). Interestingly, Gibson *et al* observed a distinction between well-differentiated keratinocytes and poorly differentiated SCC4 cells with respect to calcium ion-induced involucrin expression. In particular, high calcium concentration induced involucrin mRNA and protein in keratinocytes but not in poorly differentiated SCC4 cells (Gibson *et al*, 1996). This similarity between calcium and hypoxia regulation might be important for understanding how hypoxia induces involucrin. For example, Salnikow *et al* (2002) have described an HIF-1*α* independent pathway for the hypoxia induction of AP-1 regulated genes in which hypoxia-induced intracellular calcium release and subsequent interaction at AP-1 promoter sites are key events. Calcium ion concentration is known to increase in the outer layers of stratified epithelia (Denda *et al*, 2000) and it is conceivable that hypoxia interacts with calcium ions to stimulate the production of involucrin and other AP-1-dependent proteins. Experiments are underway to test whether the effect hypoxia on involucrin induction is direct or one mediated by intracellular calcium ions. It should be noted that the process of differentiation itself is initiated in the well-oxygenated basal cells of stratified epithelia (Watt, 1983) and is unlikely, therefore, to be initiated by hypoxia.

Involucrin induction has a time course similar to that for pimonidazole binding but is distinguished by a very steep dependence on *p*O_2_. A similarly steep dependence has been reported for VEGF mRNA induction in a number of cell lines (Chiarotto and Hill, 1999). This steepness of response is reminiscent of synergistic interactions and it is tempting to speculate that these might involve interactions between hypoxia and calcium ions reported for the case of VEGF (Salnikow *et al*, 2002). Involucrin induction is further distinguished from pimonidazole binding in that the *K*_m_ (15 mmHg) is similar to that for VEGF (Leith and Michelson, 1995; Chiarotto and Hill, 1999) and, therefore, ca 40 times higher than that for pimonidazole binding (0.4 mmHg). The rapid rate of induction, steep *p*O_2_ dependence and high *K*_m_ could be important in understanding immunostaining patterns for involucrin in squamous cell carcinomas.

In well-differentiated tumours, immunostaining for involucrin is more extensive than that for pimonidazole binding and in some areas, involucrin is expressed in the absence of pimonidazole binding (Figure 4A and B; Azuma *et al*, 2003). Superficially, a 40-fold difference in *K*_m_ for involucrin induction and pimonidazole binding might account for this. However, it does not explain why immunostaining for involucrin more closely matches pimonidazole binding in moderately differentiated tumours (Figure 4C and D) where the extent of immunostaining differs by a factor of only ca 1.5. A similar small factor of ca 2 has been reported for the difference between the extent of CAIX expression and pimonidazole binding in squamous cell carcinomas (Olive *et al*, 2001). One explanation is that different levels of acute hypoxia exist in the two tumours (Pigott *et al*, 1996). That is, the more extensive immunostaining for involucrin is due to rapid induction during acute changes in hypoxia that pimonidazole binding cannot match. However, both pimonidazole binding and involucrin are easily detected within 2 h of hypoxic exposure *in vitro* making this explanation less likely. A second possibility is that hypoxia developed during the time between pimonidazole washout (plasma *t*_1/2_=ca 5 h) and tumour biopsy, but this would require a major change in oxygen distribution in the tumour depicted in Figure 4A and B, which seems unlikely.

A third possible explanation for immunostaining patterns in Figure 4 is that oxygen gradients are steeper in moderately differentiated tumours than in well-differentiated tumours. Steep oxygen gradients would foreshorten the distance over which divergent *K*_m_s are traversed, possibly reducing the distance to the two or three cell diameters observed in moderately differentiated tumours (Figure 4C and D) and in normal tissues such as the liver and kidney (Arteel *et al*, 1995; Zhong *et al*, 1998). Conversely, shallow gradients would increase the distance over which divergent *K*_m_s are traversed allowing for involucrin induction in advance of pimonidazole binding as appears to be the case in Figure 4A and B. Variations in the slope of oxygen gradients have been proposed to account for the lack of nitroimidazole binding around areas of necrosis in a subset of glioma xenografts (Parliament *et al*, 1997; Franko *et al*, 1998; Turcotte *et al*, 2002), but it remains to be seen whether this occurs among subsets of squamous cell carcinomas.

Poorly differentiated tumours express very little involucrin even in the areas of extensive pimonidazole binding. This is observed for squamous cell carcinomas of the head and neck (Figure 4E and F) and uterine cervix (Azuma *et al*, 2003). While this does not appear to be consistent with oxygen regulation, it matches *in vitro* data where involucrin is induced by hypoxia in moderately differentiated SCC9 cells but not in poorly differentiated SCC4 cells. While there is no basis for believing that the mechanism that prevents involucrin induction in SCC4 cells is exactly the same as that which prevents involucrin expression in the hypoxic regions of poorly differentiated squamous cell carcinomas, the model system does show that dedifferentiation can suppress involucrin induction by hypoxia.

Hypoxia inhibits differentiation in some cell lines (Sahai *et al*, 1997) and possibly in breast carcinomas (Helczynska *et al*, 2003), but the association between hypoxia and involucrin expression (Figures 1, 3 and 4) indicates that this might not be true for squamous cell carcinomas. It is important to emphasise, however, that involucrin is an early marker for terminal differentiation so that tumour hypoxia, while not totally inhibiting differentiation, might arrest it at some point short of end stage differentiation. Cell lines derived from a poorly differentiated squamous cell carcinoma, for example, can express involucrin without losing proliferative capability (Auersperg *et al*, 1989). This is also true of SCC9 cells where confluent cells are easily subcultured in spite of possessing substantial levels of involucrin. Clearly, the presence of involucrin need not be a sign of end stage differentiation and further work will be required to define the extent to which hypoxic cells in squamous cell carcinomas are differentiated. This could be important because it is known that differentiation increases radiosensitivity in human carcinoma cells under both hypoxic and aerobic conditions (Hallows *et al*, 1988; Hoffmann *et al*, 1999). Radiosensitisation might be due to inhibited DNA repair arising from the limited access of DNA repair machinery in differentiated cells (Wheeler and Wierowski, 1983). To the extent that the *in vitro* results apply clinically, hypoxic cells that are more differentiated might be less radioresistant than otherwise thought.

A recurring theme in the study of endogenous hypoxia markers – whether it be HIF-1*α*, CAIX, Glut-1 or involucrin – is heterogeneity of expression (Olive *et al*, 2001; Wiesener *et al*, 2001; Haugland *et al*, 2002; Janssen *et al*, 2002; Kaanders *et al*, 2002; Airley *et al*, 2003). The basis for heterogeneity in the case of involucrin appears to be related to cell differentiation. In the case of HIF-1*α*, functional inactivation of the von Hippel Lindau factor might be the most important factor (Wiesener *et al*, 2001; Haugland *et al*, 2002; Janssen *et al*, 2002; Turner *et al*, 2002). Endogenous ORPs appear to be useful as hypoxia markers in normal tissues (e.g. Lee *et al*, 2001), but without a good understanding of the factors that control ORP expression, heterogeneity of expression of proteins such as involucrin will limit their scope as markers of human tumour hypoxia.
